# Genetic Diversity Assessment of the International Maize and Wheat Improvement Center and Chinese Wheat Core Germplasms by Non-Denaturing Fluorescence In Situ Hybridization

**DOI:** 10.3390/plants11111403

**Published:** 2022-05-25

**Authors:** Manyu Yang, Zujun Yang, Wuyun Yang, Ennian Yang

**Affiliations:** 1 Crop Research Institute, Sichuan Academy of Agricultural Sciences, Chengdu 610066, China; yangmanyu19861225@163.com (M.Y.); yangwuyun@126.com (W.Y.); 2 Key Laboratory of Wheat Biology and Genetic Improvement on Southwestern China (Ministry of Agriculture and Rural Affairs of P.R.C.), Chengdu 610066, China; 3 Environment-friendly Crop Germplasm Innovation and Genetic Improvement Key Laboratory of Sichuan Province, Chengdu 610066, China; 4 School of Life Science and Technology, University of Electronic Science and Technology of China, Chengdu 610054, China; yangzujun@uestc.edu.cn

**Keywords:** wheat, ND-FISH, genetic diversity, chromosomal translocation

## Abstract

Germplasm is the material basis for crop genetic improvement and related basic research. Knowledge of genetic diversity present in wheat is the prerequisite for wheat breeding and improvement. Non-denaturing fluorescence in situ hybridization (ND-FISH) is a powerful tool to distinguish chromosomal polymorphisms and evaluate genetic diversity in wheat. In this study, ND-FISH using Oligo-pSc119.2-1, Oligo-pTa535-1, and Oligo-(GAA)_7_ as probes were used to analyze the genetic diversity among 60 International Maize and Wheat Improvement Center (CIMMYT) derived wheat lines, and 93 cultivated wheat and landraces from the Chinese wheat core germplasm. A total of 137 polymorphic FISH patterns were obtained, in which 41, 65, and 31 were from A-, B-, and D-genome chromosomes, respectively, indicating polymorphism of B-genome > A-genome > D-genome. In addition, 22 and 51 specific FISH types were observed in the two germplasm resource lines. Twelve types of rearrangements, including seven new translocations, were detected in all 153 wheat lines. Genetic relationships among 153 wheat lines were clustered into six groups. Our research provides cytological information for rational utilization of wheat germplasm resources.

## 1. Introduction

Germplasm is the material basis of human survival and innovation of agricultural science and technology, as well as the material basis for crop genetic improvement and related basic research. Each breakthrough in crop breeding is closely related to the discovery and utilization of novel germplasm. Dwarfing genes were introduced into wheat and rice breeding, triggering the first “Green Revolution” from the 1940s to the 1960s, making an outstanding contribution to the improvement of high-yielding varieties worldwide [1,2,3]. “Taigu male-sterile wheat”, a natural mutant of dominant male sterility, is a breakthrough in providing a new method for the creation of wheat germplasm resources with great utilization value [4]. “Chuanmai42”, derived from synthetic wheat, has raised the yield of wheat to a new level in Sichuan [5]. “Sumai 3”, the most useful resistance resource against Fusarium head blight, has also played an important role in breeding disease-resistant wheat worldwide [6,7]. Therefore, the utilization of germplasm is of great significance to breeding breakthrough varieties, promoting the sustainable development of the modern seed industry, and guaranteeing national food security.

Common wheat (*Triticum aestivum* L.), one of the most important food crops for humanity, feeds 36% of the world population [8]. Unfortunately, the genetic base of wheat breeding is shrinking due to most breeding programs relying on a few parents in developing germplasm pools [9,10]. Autrique et al. [11] found that the same 15 ancestors were present in the pedigree of at least 80% of all 54 wheat cultivars, with five being found in all of them. In a study of wheat genetic diversity trends during domestication and breeding, a loss of genetic diversity was also observed from landrace cultivars to the elite breeding germplasm [12]. Such narrow genetic diversity is a challenge for sustainable wheat production with the changing climate and rapidly growing world population [13]. Landraces, wild species, and wild relatives possess high levels of genetic diversity for valuable traits, which may have been lost in elite genetic pools by selective processes [14]. Studies have shown that the genetic diversity could be increased by the introgression of novel lines from landrace cultivars, which contained numerous unique alleles that were absent in modern bread wheat cultivars [12]. Besides, foreign germplasm resources, such as the CIMMYT germplasm, have played an important role in wheat breeding and improvement, and the introduction of these resources has enriched the genetic diversity of wheat germplasm [15]. Thus, the utilization of landraces and the continuous introduction of foreign germplasm are of great significance to wheat breeding.

Collection and utilization of wheat germplasm are essential for the improvement of wheat cultivars, but genetic diversity present in wheat germplasm is the prerequisite for the utilization of germplasm. Currently, several methods are widely used to evaluate the genetic diversity of wheat, including morphological markers [13], biochemical markers [16], and molecular markers [17,18,19]. In addition, cytogenetic approaches such as C-banding and fluorescent in situ hybridization (FISH) have also succeeded in revealing considerable polymorphism within the wheat genome [20], except for the identification of alien introgressions in wheat background [21,22] and studying chromosome behavior [23]. In recent years, ND-FISH based on oligonucleotide probes has been developed to detect wheat chromosomes rapidly, accurately, and at scale. Tang et al. [24] and Fu et al. [25] first developed the oligonucleotide probes Oligo-pTa535 and Oligo-pSc119.2 for ND-FISH, which can replace the repetitive DNA sequences pSc119.2 and pAs1 to identify the 21 wheat chromosomes. Since then, the technology has been widely used as a powerful karyotyping tool to identify wheat chromosome variations. Jiang et al. [26] constructed the chromosome karyotype for 85 common wheat varieties, displayed by ND-FISH using Oligo-pSc119.2-1, Oligo-pTa535-1, and Oligo-(GAA)_6_ as probes. Guo et al. [27] reported the genetic diversity of 76 representative Chinese wheat lines by ND-FISH. Ren et al. [28] performed ND-FISH using five oligonucleotide probes to examine 21 wheat cultivars and lines. Hu et al. [29] assessed chromosome polymorphisms of 166 common wheat cultivars using 11 oligonucleotide probes. However, studies for evaluating the Chinese and CIMMYT wheat core germplasm by ND-FISH have not been reported.

The CIMMYT Mexican core germplasm (CIMCOG) has facilitated wheat breeding for grain yield and quality worldwide, especially in warm areas [30]. Several studies have been performed to either evaluate or use this germplasm for slow rusting resistance [31], lodging-related traits [32], seed storability [33], seed dormancy and longevity [34], grain quality [35], and agronomic traits [36]. Studies have also been conducted on resistance to stripe rust and powdery mildew of Chinese core germplasm [37,38]. In the present study, 60 CIMCOG wheat lines and 93 Chinese applied core germplasm (CACG) wheat lines were used to assess genetic diversity by ND-FISH using Oligo-pSc119.2-1, Oligo-pTa535-1, and Oligo-(GAA)_7_ as probes, in order to provide cytological information for improvements in wheat breeding.

## 2. Results

### 2.1. Construction of Wheat Karyotype by ND-FISH

**Figure 1:**
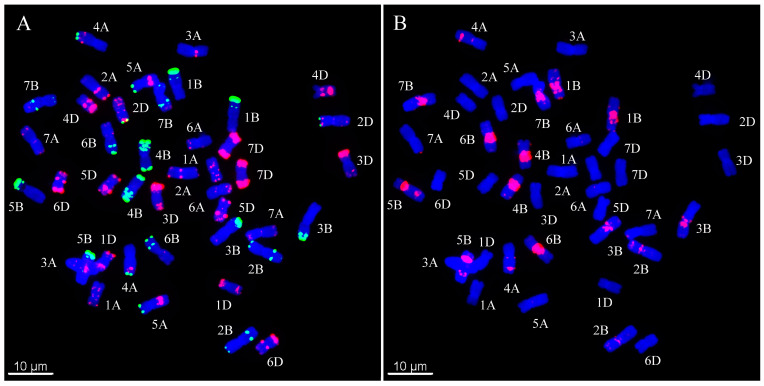
Standard ND-FISH pattern of Chinese Spring. (**A**) Oligo-pTa535 (red) and Oligo-pSc119.2 (green) were used as FISH probes. (**B**) Oligo-(GAA)_7_ (red) were used as FISH probes. Chromosomes were counterstained with DAPI (blue). Scale bar indicates 10 μm.

**Figure 2:**
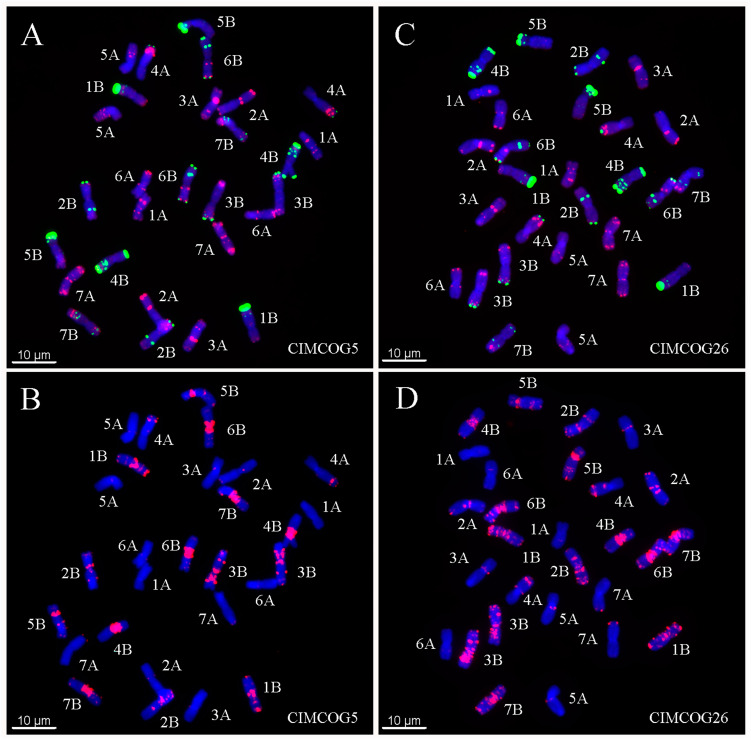
ND-FISH of the lines CIMCOG5 (**A**,**B**) and CIMCOG26 (**C**,**D**) in the CIMMYT Mexican wheat core germplasm. (**A**,**C**) Oligo-pTa535 (red) and Oligo-pSc119.2 (green) were used as FISH probes. (**B**,**D**) Oligo-(GAA)_7_ (red) were used as FISH probes. Chromosomes were counterstained with DAPI (blue). Scale bar indicates 10 μm.

Five seeds were randomly selected from each line, and the metaphase chromosomes of each seed were analyzed by ND-FISH with Oligo-PSC119.2-1, Oligo-pTa535-1, and Oligo-(GAA)_7_. The standard karyotype of Chinese Spring (CS) was established based on the hybridization patterns of the three probes. As shown in Figure 1, the Oligo-pSc119.2 probe mainly hybridized with B-genome chromosomes, whereas the hybridization signals of Oligo-pTa535-1 were mainly distributed on A- and D-genome chromosomes. The Oligo-(GAA)_7_ probe produced strong signals at most peri-centromeric regions for all the B-genome chromosomes analyzed. Using the karyotype of CS as reference, two of 60 CIMCOG wheat lines were tetraploid (2n = 28) (Figure 2, Table 1), whereas 58 of 60 CIMCOG wheat lines and all 93 CACG wheat lines were hexaploid (2n = 42) (Table 1 and Table 2). Therefore, combined with the three probes, we were able to distinguish all 21 wheat chromosomes and determine the hybrid signal polymorphism and chromosomal rearrangement among the wheat lines by comparing the distribution of FISH patterns. 

### 2.2. ND-FISH Polymorphisms of A-, B-, and D-Genome Chromosomes

**Figure 3:**
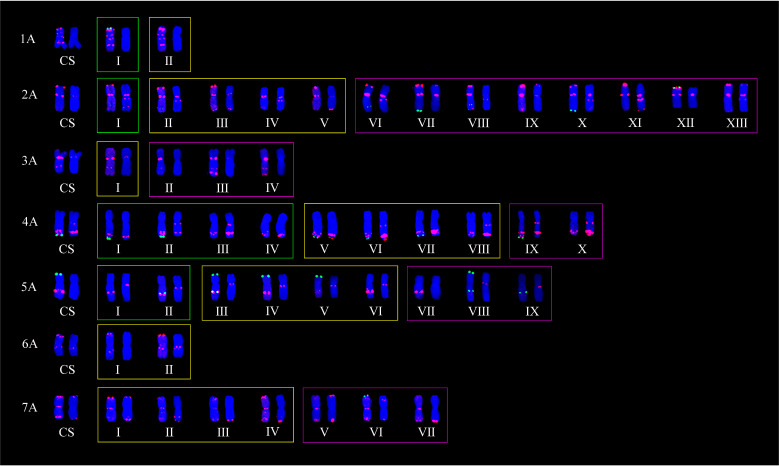
Polymorphic FISH types of A-genome. CS represents Chinese Spring. Oligo-pTa535 (red) and Oligo-pSc119.2 (green) were used as probes on the left chromosomes, while Oligo-(GAA)_7_ (red) was used as the probe on the right chromosomes. Yellow boxes represent shared types, green boxes represent specific types in the CIMCOG wheat lines, and purple boxes represent specific types in the CACG wheat lines.

**Figure 4:**
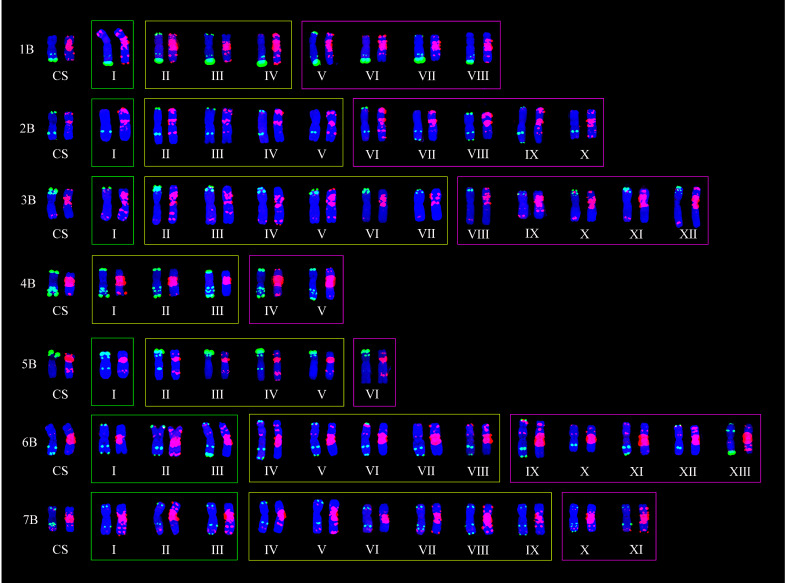
Polymorphic FISH types of B-genome. CS represents Chinese Spring. Oligo-pTa535 (red) and Oligo-pSc119.2 (green) were used as probes on the left chromosomes, while Oligo-(GAA)_7_ (red) was used as the probe on the right chromosomes. Yellow boxes represent shared types, green boxes represent specific types in the CIMCOG wheat lines, and purple boxes represent specific types in the CACG wheat lines.

**Figure 5:**
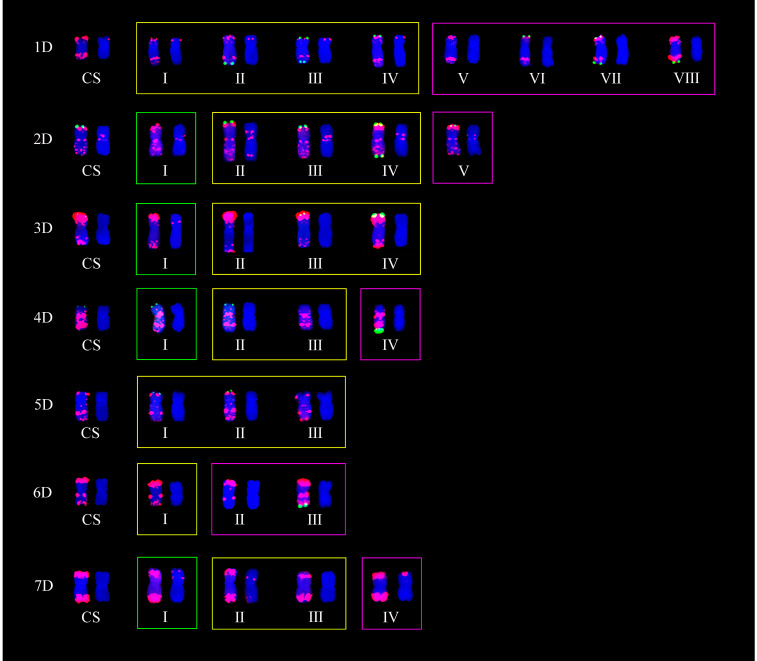
Polymorphic FISH types of D-genome. CS represents Chinese Spring. Oligo-pTa535 (red) and Oligo-pSc119.2 (green) were used as probes on the left chromosomes, while Oligo-(GAA)_7_ (red) was used as the probe on the right chromosomes. Yellow boxes represent shared types, green boxes represent specific types in the CIMCOG wheat lines, and purple boxes represent specific types in the CACG wheat lines.

Based on the comparison of ND-FISH hybridization signals displayed by the three probes, polymorphisms of wheat chromosomes could be displayed by the variation of tandem repeats in the amount and distribution. In the 153 lines, 137 polymorphic FISH types among 21 chromosomes were obtained (Table 3); 41 from A-genome (Figure 3), 65 from B-genome (Figure 4), and 31 from D-genome chromosomes (Figure 5). The number of FISH types ranged from 2 to 13, 5 to 13, and 3 to 8 in A-, B-, and D-genomes, respectively. All 21 chromosomes had two types of ND-FISH hybridization signal patterns at least. Among these, chromosomes 1A and 6A only had two types, whereas chromosomes 2A, 4A, 2B, 3B, 6B, and 7B had more than 10 types. Chromosomes 2A and 6B had the highest number of types. Separately, the 60 CIMCOG wheat lines contained 91 polymorphic types, including 28, 41, and 22, respectively, from A-, B-, and D-genome chromosomes. The 93 CACG wheat lines had 137 types, including 39, 55, and 27 from A-, B-, and D-genome chromosomes, respectively. In other words, the polymorphism of ND-FISH hybridization signals in genomes was B-genome > A-genome > D-genome. Therefore, ND-FISH based on different oligonucleotide probes could generate sufficient hybridization signal patterns to evaluate the genetic diversity of wheat.

Figure 3, Figure 4 and Figure 5 show that all 21 chromosomes of the CIMCOG and CACG wheat lines contained common FISH types (yellow boxes). Furthermore, the two germplasm resources also had their own specific FISH types. For example, the CIMCOG wheat lines had 22 specific FISH types (green boxes), among which chromosomes 3A, 6A, 7A, 4B, 1D, 5D, and 6D had no specific type; chromosomes 1A, 2A, 1B, 2B, 3B, 5B, 2D, 3D, 4D, and 7D had one specific type; and chromosome 5A possessed two specific types, chromosomes 6B and 7B contained three specific types, and chromosome 4A contained four specific types. Similarly, 51 specific types were observed in the CACG wheat lines: no specific types from chromosomes 1A, 6A, 3D, and 5D; one specific type from chromosomes 5B, 2D, 4D, and 7D; two specific types from chromosomes 4A, 4B, 7B, and 6D; three specific types from chromosomes 3A, 5A, and 7A; four specific types from chromosomes 1B and 1D; five specific types from chromosomes 2B, 3B, and 6B; and eight specific types from chromosome 1A. These results showed that the genetic diversity of the CACG wheat lines was richer than that of the CIMCOG wheat lines.

### 2.3. Detection of Chromosomal Translocations

**Figure 6:**
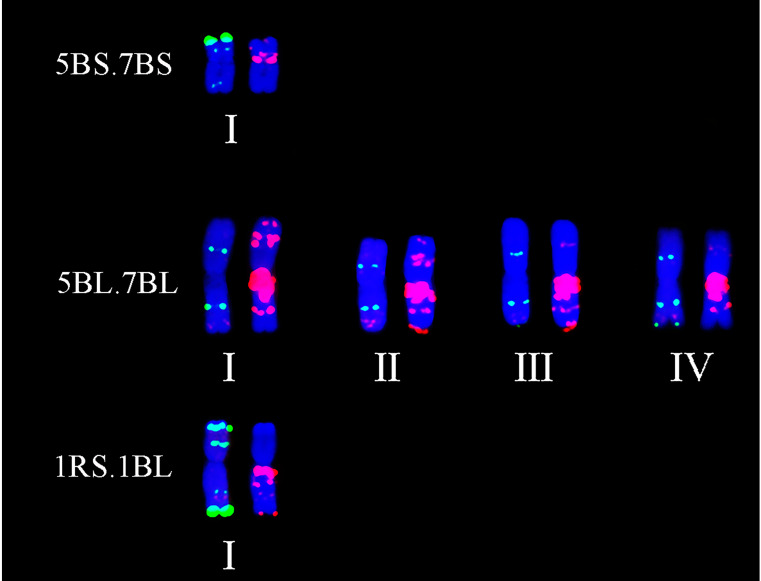
Polymorphic FISH types of translocation chromosomes. Oligo-pTa535 (red) and Oligo-pSc119.2 (green) were used as probes on the left chromosomes, while Oligo-(GAA)_7_ (red) was used as the probe on the right chromosomes.

**Figure 7:**
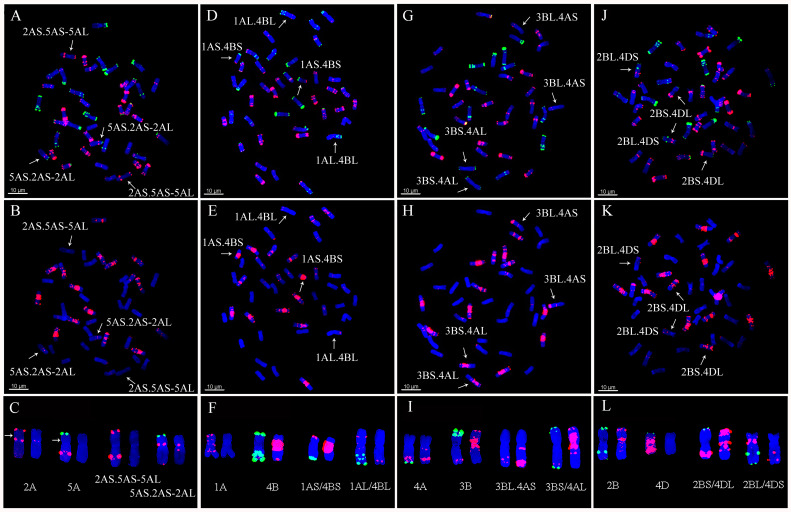
ND-FISH pattern of translocation chromosomes of the lines CACG4 (**A**–**C**), CACG6 (**D**–**F**), CACG12 (**G**–**I**), CACG23 (**J**–**L**) in the Chinese applied core germplasm. (**A**,**D**,**G**,**J**) Oligo-pTa535 (red) and Oligo-pSc119.2 (green) were used as FISH probes. (**B**,**E**,**H**,**K**) Oligo-(GAA)_7_ (red) was used as a FISH probe. Chromosomes were counterstained with DAPI (blue). Arrows show translocation chromosomes. Scale bar indicates 10 μm.

**Figure 8:**
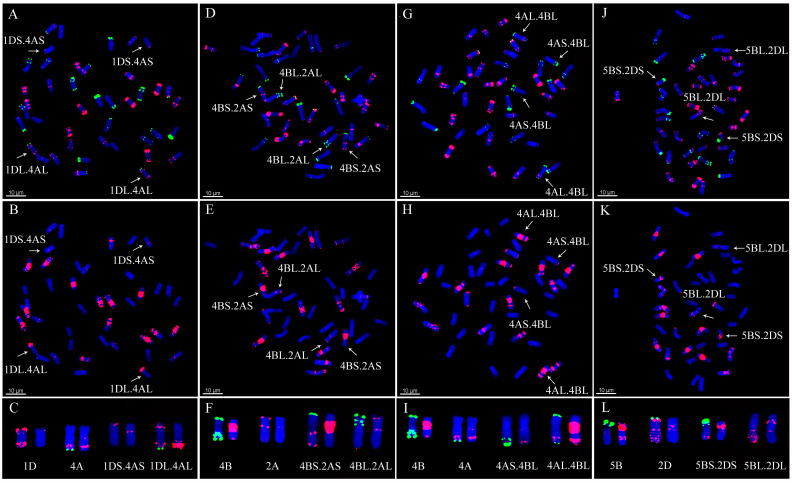
ND-FISH pattern of translocation chromosomes of the lines CACG28 (**A**–**C**), CACG68 (**D**–**F**), CACG69 (**G**–**I**), CACG84 (**J**–**L**) in the Chinese applied core germplasm. (**A**,**D**,**G**,**J**) Oligo-pTa535 (red) and Oligo-pSc119.2 (green) were used as FISH probes. (**B**,**E**,**H**,**K**) Oligo-(GAA)7 (red) was used as FISH probe. Chromosomes were counterstained with DAPI (blue). Arrows show translocation chromosomes. Scale bar indicates 10 μm.

Twelve types of chromosomal translocations were found among all the 153 lines using ND-FISH, in which the CIMCOG wheat lines contained three types (Table 1) and the CACG wheat lines had nine types (Table 2). 1RS.1BL translocation was observed in 12 lines, including CIMCOG14, CIMCOG38, CIMCOG43, CIMCOG45, CIMCOG46, CACG17, CACG33, CACG37, CACG41, CACG78, CACG79, and CACG80. Three lines, CIMCOG8, CIMCOG42, and CIMCOG53, contained 5BS.7BS/5BL.7BL translocation, while two lines, CIMCOG17 and CIMCOG59, possessed 1RS.1BL and 5BS.7BS/5BL.7BL translocations. Four polymorphic FISH types were detected in the 5BL.7BL translocation chromosome, but only one type in the 1RS.1BL and 5BS.7BS translocation chromosomes (Figure 6). Furthermore, eight Robertsonian translocations, including 1RS.7DS/1BL.7DL, 1AS.4BS/1AL.4BL, 3BS.4AL/3BL.4AS, 2BL.4DS/2BS.4DL, 1DL.4AL/1DS.4AS, 4BS.2AS/4BL.2AL, 4AS.4BL/4AL.4BS, and 5BS.2DS/5BL.2DL were found in lines CACG39, CACG6, CACG12, CACG23, CACG28, CACG68, CACG69, and CACG84, respectively (Figure 7 and Figure 8). Non-Robertsonian translocations, 2AS.5AS-5AL/5AS.2AS-2AL, between 2A and 5A chromosomes were also observed in CACG4 (Figure 7A–C). Based on the signal patterns, the breakpoints were located in the short arm of chromosome 2A distal to the telomeric signals of Oligo-pTa535-1 and the short arm of chromosome 5A distal to the telomeric signals of Oligo-pSc119.2. These translocations involved chromosomes 1A, 2A, 4A, 5A, 1B, 2B, 3B, 4B, 5B, 7B, 1D, 2D, and 4D, among which 4A and 4B had the largest number of translocations, each with three translocations. However, these translocation chromosomes occurred only in one line with no polymorphism. These results indicated that it could effectively identify chromosome rearrangement of A-, B-, and D-genome in wheat when using Oligo-pTa535, pSc119.2-1-1, and Oligo-(the GAA)_7_.

### 2.4. Distributions of Different Chromosomal Types between the Two Wheat Germplasm Resources

The distributions of different types in A-, B-, and D-genome chromosomes are listed in Table 3. Types 1A^I^, 2A^III^, 3A^I^, 4A^II^, 5A^IV^, 6A^I^, 7A^I^, 1B^III^, 2B^II^, 3B^III^, 4B^I^, 5B^II^, 6B^IV^, 7B^IV^, 1D^I^, 2D^III^, 3D^II^, 4D^II^, 5D^I^, 6D^I^, 7D^II^ occurred most frequently in the CIMCOG wheat lines when types 1A^II^, 2A^III^, 3A^I^, 4A^IX^, 5A^IV^, 6A^II^, 7A^IV^, 1B^II^, 2B^III^, 3B^V^, 4B^I^, 5B^II^, 6B^VIII^, 7B^V^, 1D^I^, 2D^III^, 3D^III^, 4D^II^, 5D^III^, 6D^I^, 7D^II^ appeared at high frequencies in the CACG wheat lines. Among them, types 2A^III^, 3A^I^, 5A^IV^, 4B^I^, 5B^II^, 1D^I^, 2D^III^, 4D^II^, 6D^I^, and 7D^II^ were the main types in both the CIMCOG wheat lines and the CACG wheat lines, accounting for a relatively high proportion.

### 2.5. Genetic Relationships Revealed by FISH Patterns

**Figure 9:**
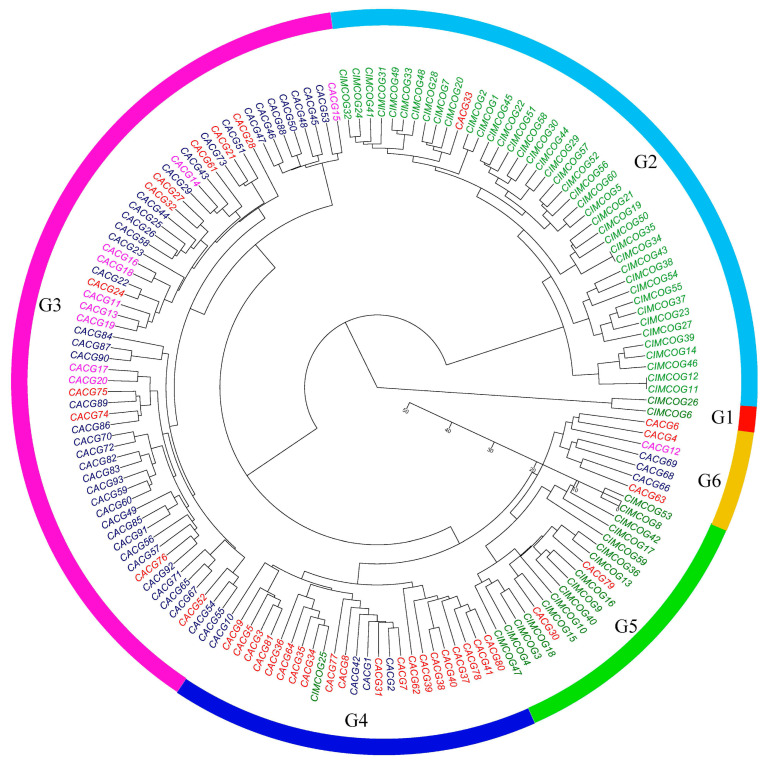
Genetic relationships among 153 wheat lines revealed by FISH patterns. Green letters show the CIMMYT Mexican core germplasm. Red, purple, and blue letters show breeding varieties, introduced varieties, and landraces in the Chinese applied core germplasm.

Based on the polymorphism of the FISH types, a dendrogram was constructed by cluster analysis among the 153 wheat lines. These wheat lines were divided into six groups (G1–G6) (Figure 9). The two tetraploid wheat lines derived from CIMCOG were in G1; the other CIMCOG wheat lines were mainly distributed in G2 and G5, with only CIMCOG25 in G4. Ninety of the CACG wheat lines were dispersed in G3, G4, and G6, except for three lines in G2 and G5. Among the CACG wheat lines, Chinese landraces and introduced varieties were mainly in G3, while breeding varieties were in G4.

## 3. Discussion

The ND-FISH technique not only accurately identified the chromosomes of A-, B-, and D-genomes in wheat but also analyzed wheat chromosomal diversity based on oligonucleotide probes showing different signal patterns on the same chromosome of different lines. In this study, all 21 wheat chromosomes were distinguished by ND-FISH using Oligo-pSc119.2-1, Oligo-pTa535-1, and Oligo-(GAA)_7_, and the standard karyotype of CS was established, which could accurately detect the variation of chromosomes. The polymorphisms of chromosomes of 85 wheat cultivars/lines were investigated by ND-FISH analysis using Oligo-pSc119.2-1, Oligo-pTa535-1, and Oligo-(AAG)_6_ as probes. The results showed that each line had a unique ND-FISH karyotype, and more variations of wheat chromosomes were displayed [26]. Seventy-six representative Chinese wheat lines were selected for investigation by ND-FISH using Oligo-pTa535, Oligo-pSc119.2, and Oligo-(GAA)_8_ as probes, and the number of FISH types ranged from 2 to 7, 2 to 6, and 1 to 5 in A-, B-, and D-genome chromosomes, respectively [27]. Novel karyotype characteristics of 166 common wheat cultivars bred from the hometown CS were revealed using 11 oligonucleotide probes [29]. Chromosomes 5A, 3B, and 1D showed the highest number of karyotype variations, which were 24, 18, and 6, respectively [29]. Moreover, the genetic diversity of Asian and European common wheat lines was assessed by pTa535 and pSc119.2 probes, and the ranges of the number of FISH types in the A-, B-, and D-genomes were 2 to 8, 3 to 7, and 2 to 4, respectively, in which the FISH types of the 5A chromosome occurred at the highest frequency [39]. In the present study, ND-FISH using Oligo-pSc119.2-1, Oligo-pTa535-1, and Oligo-(GAA)_7_ as probes were used to analyze the genetic diversity between the CIMCOG and CACG wheat lines. ND-FISH reflected that 97.39% of all 153 wheat lines could be clearly recognized except CIMCOG11, CIMCOG12, CIMCOG34, and CIMCOG35. A total of 137 polymorphic FISH types were obtained, of which 41, 65, and 31 were from A-, B-, and D-genome chromosomes, respectively. All 21 chromosomes had at least two types of ND-FISH hybridization signal patterns. Chromosomes 2A and 6B had the highest number (13) of types. Besides, we found polymorphism of B-genome > A-genome > D-genome in all 153 lines, as well as in the CACG and CIMCOG wheat lines alone. The same phenomenon was found by Guo et al. [27] and Yang et al. [39]. Recently, Hu et al. [29] also found that the FISH types of the D-genome were less than those of A- and B-genomes. These results analyzed by ND-FISH were consistent with the distribution of AFLP markers [40], microsatellite markers [41], DArT markers [42], and GBS-SNPs markers [43,44] in the three genomes, respectively. Therefore, ND-FISH is an effective tool for distinguishing chromosomal types and estimating genetic diversity in wheat.

Chromosomal translocations are large-scale mutational events that play an evolutionary role in intra-specific divergence and speciation [45]. Identification of chromosomal translocations is necessary for a full understanding of germplasm resources. In this study, 12 types of complex translocations were detected among 153 wheat lines according to the ND-FISH analysis. For instance, the 1RS.1BL translocation was observed in 14 wheat lines of both CIMCOG and CACG wheat lines, accounting for 9.15%. Because of the superior genes for grain yield and stress tolerance in the 1RS chromosome, the 1RS.1BL translocation is widely used in wheat breeding [46]. Previous studies revealed the polymorphism of 1BL.1RS using probe (AAG)_n_ [27,29]. However, no polymorphism of 1BL.1RS was detected in this study. Based on the pedigree analysis, the origin of the 1BL.1RS translocation might be single. For example, CACG79 yunmai34, derived from the cross Kavkaz/IRN68-77 and CACG17 Lovrin10 are both derivatives of Neuzucht. CACG37 shannong7859 was derived from Predgornia 2. 1RS.7DS/1BL.7DL translocations were only detected in CACG39 Lumai1, which was derived from Aifeng 3//Mengxian 201/Neuzucht. Qi et al. [47,48] explained that this pair of translocations were produced by spontaneous chromosome translocation between 1BL.1RS and 7DS.7DL during hybridization. 5BS.7BS/5BL.7BL translocations were observed in five lines, in which the 5BL.7BL translocation chromosome had four FISH signal patterns. The four patterns might have occurred independently in these lines based on pedigree. 5BS.7BS/5BL.7BL translocations were widely found in wheat from western countries [49,50,51]. However, in China, the related reports only mention Sichuan varieties with French ancestry [29,52,53]. In addition, the translocations were only in the CIMCOG wheat lines in this study. 5BS.7BS/5BL.7BL translocations might be related to the adaptation to specific environmental conditions. A total of seven Robertsonian translocations were found, including 1AS.4BS/1AL.4BL, 3BS.4AL/3BL.4AS, 2BL.4DS/2BS.4DL, 1DL.4AL/1DS.4AS, 4BS.2AS/4BL.2AL, 4AS.4BL/4AL.4BS, and 5BS.2DS/5BL.2DL. 4BS.2AS/4BL.2AL translocations widely existed in Ethiopian tetraploid wheat, and the fixation of the translocations indicated the presence of a severe bottleneck during its dispersal [54]. However, 4BS.2AS/4BL.2AL translocations in this study were found in hexaploid wheat; thus, they might be different from previous findings. The remaining six chromosomal translocations were new translocations that have not been reported before. 2AS.5AS-5AL/5AS.2AS-2AL non-Robertsonian translocations observed in Kefeng3 were also new translocations. All the translocations involved chromosomes 1A, 2A, 4A, 5A, 1B, 2B, 3B, 4B, 5B, 7B, 1D, 2D, and 4D, among which 4A and 4B were involved in more rearrangements than the others. In addition, 6, 8, and 4 translocations involving the A-, B-, and D-genomes, respectively, were detected in this study. It appeared that the B-genome was more prone to chromosomal translocations than the A- and D-genomes, which was consistent with previous studies [39,41,50].

Several studies have found that the genetic diversity of wheat lines is lower compared with their progenitors [13,19,55,56]. Utilization of wheat core germplasm resources and continuous introduction of foreign germplasm will play an important role in the enrichment of genetic diversity. In this study, the diversity of the CACG wheat lines (137 types) was more than the CIMCOG wheat lines (91 types). Except for common FISH types, most of the 21 chromosomes between the CIMCOG and CACG wheat lines contained their own specific FISH types. About 22 specific FISH types were detected in the CIMCOG wheat lines, while 51 specific FISH types were found in the CACG wheat lines. The specific FISH types were also significantly higher in the CACG wheat lines than in the CIMCOG wheat lines. The CIMCOG wheat lines mainly consisted of modern breeding varieties (lines), but the CACG wheat lines included not only breeding varieties but also introduced varieties and landraces. Wheat landraces contain wider genetic diversity than most breeding programs [10]. Therefore, the diversity of species composition might be responsible for more specific FISH types in the CACG wheat lines. Furthermore, based on the distributions of different types, 10 common types, such as 2A^III^, 3A^I^, 5A^IV^, 4B^I^, 5B^II^, 1D^I^, 2D^III^, 4D^II^, 6D^I^, and 7D^II^, occurred most frequently in both CIMCOG and CACG wheat lines. Only three specific types were predominant in their respective populations, 1A^I^ and 4A^II^ in the CIMCOG wheat lines, while 4A^IX^ in the CACG wheat lines. Previous studies indicated that chromosome karyotype was closely associated with adaptation [27,29], explaining why these chromosome types appear more frequently in their respective populations. 

The contribution of the CIMMYT germplasm to Chinese wheat varieties is significant. The proportion of varieties with the CIMMYT germplasm increased from less than 10% in the early 1980s to nearly 25% by 2011 [57]. The application of the CIMMYT germplasm significantly enhanced the performance of Chinese varieties in important traits, including yield potential, processing quality, disease resistance, and early maturity [57]. Thus, it is essential to fully understand the CIMMYT germplasm for breeding. For instance, promising slow rusting resistance was observed in CIMCOG14, 17, 38, 43, 46, 53, and 59 [31]. The cytological results of this study showed that CIMCOG14, 38, 43, and 46 contained 1BL.1RS translocation, CIMCOG53 carried 5BS.7BS/5BL.7BL translocations, and CIMCOG17 and 59 possessed 1BL.1RS and 5BS.7BS/5BL.7BL translocations. The same was true for the CACG wheat lines. CACG80, Xingyi 4, immune to stem rust, stripe rust, and powdery mildew [38], was a 1BL.1RS translocation line. CACG69, Shanmai, showing resistance to both stem rust and stripe rust [38], carried 4AS.4BL/4AL.4BS translocations. All of these results can provide useful information for the rational utilization of wheat germplasm.

## 4. Materials and Methods

### 4.1. Plant Materials

A total of 154 lines, including 60 CIMCOG wheat lines (Table 1), 93 CACG wheat lines, and CS (Table 2), were investigated in this study. The CACG wheat lines consisted of 36 breeding varieties, 10 introduced varieties, and 47 Chinese landraces. The seeds were provided by Crop Research Institute, Sichuan Academy of Agricultural Sciences, Sichuan, China.

### 4.2. ND-FISH Analysis

Five seeds were randomly selected from each line for ND-FISH analysis. Root-tip metaphase chromosomes were prepared as described by Han et al. [58]. The oligonucleotide probes, including Oligo-pSc119.2-1, Oligo-pTa535-1, and Oligo-(GAA)_7_, were used for ND-FISH analysis. Sequences of these oligonucleotide probes were referenced from Tang et al. in Table 4 [24]. Oligo-pSc119.2-1 was 5′-end-labeled with 6-carboxyfluorescein (6-FAM, green); Oligo-pTa535-1 was 5′-end-labeled with 6-carboxytetramethylrhodamine (TAMRA, red); and Oligo-(GAA)_7_ was 5′-end-labeled with Cy5 (red). The detailed process of FISH was performed following Fu et al. [25]. Probe amounts per slide were as described by Tang et al. [24]. Each slide with the cell spread was added with 10 μL probe mixture (each probe in 2 × SSC and 1 × TE buffer, pH 7.0) and covered with a glass coverslip. Slides were stored in a moist box at 42 °C for 1 h and eluted in 2 × SSC solution at room temperature. The slides were mounted with Vectashield mounting medium (Vector Laboratories) with DAPI (4′,6-diamidino-2-phenylindole), and chromosomes were counterstained blue. Photomicrographs of FISH chromosomes were taken using an epifluorescence microscope (DM4B, Leica) and processed with Photoshop CC.

### 4.3. Data Analysis

Statistical analysis was performed using Microsoft Excel 2010. The dendrogram was constructed using http://www.bioinformatics.com.cn (accessed on 12 March 2022), an online platform for data analysis and visualization.

## 5. Conclusions

High-resolution ND-FISH facilitates the identification of chromosomal polymorphisms and structural rearrangements in wheat. A total of 137 polymorphic FISH types were obtained from the CIMCOG and CACG wheat lines, in which 41, 65, and 31 were from A-, B-, and D-genome chromosomes, respectively, indicating polymorphism of B-genome > A-genome > D-genome. In addition, 22 and 51 specific FISH types were observed in the two germplasm resources, respectively. Twelve types of rearrangements, including seven new translocations, were detected in all 153 wheat lines. Genetic relationships among 153 wheat lines were clustered into six groups. Overall, our research can provide valuable cytological information for rational utilization of wheat germplasm.

## Figures and Tables

**Table 1:** List of CIMMYT Mexican core germplasm.

Number	Name	No. of Chr	Translocation
CIMCOG1	ATTILA	2n = 42	
CIMCOG2	ATTILA∗2/PBW65	2n = 42	
CIMCOG3	ATTILA∗2/PBW65∗2/5/REH/HARE//2∗BCN/3/CROC_1/AE.SQUARROSA (213)//PGO/4/HUITES	2n = 42	
CIMCOG4	ATTILA//PGO/SERI/3/PASTOR	2n = 42	
CIMCOG5	BABAX/LR42//BABAX/3/ER2000	2n = 42	
CIMCOG6	BABAX/LR42//BABAX/3/VORB	2n = 28	
CIMCOG7	BACANORA T 88	2n = 42	
CIMCOG8	BAVIACORA M 92	2n = 42	5BS.7BS, 5BL.7BL
CIMCOG9	BCN/RIALTO	2n = 42	
CIMCOG10	BCN/WBLL1	2n = 42	
CIMCOG11	BECARD	2n = 42	
CIMCOG12	BECARD/KACHU	2n = 42	
CIMCOG13	BRBT1∗2/KIRITATI	2n = 42	
CIMCOG14	C80.1/3∗BATAVIA//2∗WBLL1/5/REH/HARE//2∗BCN/3/CROC_1/AE.SQUARROSA (213)//PGO/4/HUITES	2n = 42	1RS.1BL
CIMCOG15	SAUAL/4/CROC_1/AE.SQUARROSA (205)//KAUZ/3/ATTILA/5/SAUAL	2n = 42	
CIMCOG16	SAUAL/WHEAR//SAUAL	2n = 42	
CIMCOG17	CHIR3/4/SIREN//ALTAR 84/AE.SQUARROSA (205)/3/3∗BUC/5/PFAU/WEAVER	2n = 42	1RS.1BL, 5BS.7BS, 5BL.7BL
CIMCOG18	CHWL86/6/FILIN/IRENA/5/CNDO/R143//ENTE/MEXI_2/3/AEGILOPS SQUARROSA (TAUS)/4/WEAVER	2n = 42	
CIMCOG19	CMH79A.955/4/AGA/3/4∗SN64/CNO67//INIA66/5/NAC/6/RIALTO	2n = 42	
CIMCOG20	CIRNO C 2008	2n = 42	
CIMCOG21	CNDO/R143//ENTE/MEXI_2/3/AEGILOPS SQUARROSA (TAUS)/4/OCI/5/PASTOR/6/TEMPORALERA M 87/ROMO96	2n = 42	
CIMCOG22	CNDO/R143//ENTE/MEXI_2/3/AEGILOPS SQUARROSA (TAUS)/4/WEAVER/5/2∗KAUZ	2n = 42	
CIMCOG23	CNO79//PF70354/MUS/3/PASTOR/4/BAV92∗2/5/FH6-1-7	2n = 42	
CIMCOG24	CROC_1/AE.SQUARROSA (205)//BORL95/3/PRL/SARA//TSI/VEE#5/4/FRET2	2n = 42	
CIMCOG25	GK ARON/AG SECO 7846//2180/4/2∗MILAN/KAUZ//PRINIA/3/BAV92	2n = 42	
CIMCOG26	KINGBIRD #1//INQALAB 91∗2/TUKURU	2n = 28	
CIMCOG27	KFA/3/PFAU/WEAVER//BRAMBLING/4/PFAU/WEAVER∗2//BRAMBLING	2n = 42	
CIMCOG28	MEX94.27.1.20/3/SOKOLL//ATTILA/3∗BCN	2n = 42	
CIMCOG29	MILAN/KAUZ//PRINIA/3/BAV92	2n = 42	
CIMCOG30	MUNAL #1	2n = 42	
CIMCOG31	ATTILA/PASTOR	2n = 42	
CIMCOG32	OASIS/5∗BORL95/5/CNDO/R143//ENTE/MEXI75/3/AE.SQ/4/2∗OCI	2n = 42	
CIMCOG33	MISR 1	2n = 42	
CIMCOG34	OASIS/SKAUZ//4∗BCN/3/2∗PASTOR/5/FRET2∗2/4/SNI/TRAP#1/3/KAUZ∗2/TRAP//KAUZ/6/SAUAL #1	2n = 42	
CIMCOG35	PANDORA//WBLL1∗2/BRAMBLING	2n = 42	
CIMCOG36	PASTOR/3/URES/JUN//KAUZ/4/WBLL1	2n = 42	
CIMCOG37	PAVON F 76	2n = 42	
CIMCOG38	PBW343∗2/KUKUNA∗2//FRTL/PIFED	2n = 42	1RS.1BL
CIMCOG39	PFAU/SERI.1B//AMAD/3/WAXWING	2n = 42	
CIMCOG40	ARMENT//2∗SOOTY_9/RASCON_37/4/CNDO/PRIMADUR//HAI-OU_17/3/SNITAN	2n = 42	
CIMCOG41	QUAIU #3//MILAN/AMSEL	2n = 42	
CIMCOG42	RL6043/4∗NAC//2∗PASTOR	2n = 42	5BS.7BS, 5BL.7BL
CIMCOG43	ROLF07∗2/5/REH/HARE//2∗BCN/3/CROC_1/AE.SQUARROSA (213)//PGO/4/HUITES	2n = 42	1RS.1BL
CIMCOG44	SERI M 82	2n = 42	
CIMCOG45	SIETE CERROS T66	2n = 42	1RS.1BL
CIMCOG46	SOKOLL∗2/3/BABAX/LR42//BABAX	2n = 42	1RS.1BL
CIMCOG47	SOKOLL//PBW343∗2/KUKUNA/3/ATTILA/PASTOR	2n = 42	
CIMCOG48	TACUPETO F2001/SAUAL/4/BABAX/LR42//BABAX∗2/3/KURUKU	2n = 42	
CIMCOG49	TACUPETO F2001/BRAMBLING∗2//KACHU	2n = 42	
CIMCOG50	TC870344/GUI//TEMPORALERA M 87/AGR/3/2∗WBLL1	2n = 42	
CIMCOG51	TRAP#1/BOW/3/VEE/PJN//2∗TUI/4/BAV92/RAYON/5/KACHU #1	2n = 42	
CIMCOG52	TRCH/SRTU//KACHU	2n = 42	
CIMCOG53	UP2338∗2/4/SNI/TRAP#1/3/KAUZ∗2/TRAP//KAUZ/5/MILAN/KAUZ//CHIL/CHUM18/6/UP2338∗2/4/SNI/TRAP#1/3/KAUZ∗2/TRAP//KAUZ	2n = 42	5BS.7BS, 5BL.7BL
CIMCOG54	W15.92/4/PASTOR//HXL7573/2∗BAU/3/WBLL1	2n = 42	
CIMCOG55	BECARD	2n = 42	
CIMCOG56	WBLL1∗2/4/BABAX/LR42//BABAX/3/BABAX/LR42//BABAX	2n = 42	
CIMCOG57	WBLL1∗2/KURUKU∗2/5/REH/HARE//2∗BCN/3/CROC_1/AE.SQUARROSA (213)//PGO/4/HUITES	2n = 42	
CIMCOG58	WBLL1∗2/TUKURU∗2/4/CROC_1/AE.SQUARROSA (205)//BORL95/3/2∗MILAN	2n = 42	
CIMCOG59	WHEAR/SOKOLL	2n = 42	1RS.1BL, 5BS.7BS, 5BL.7BL
CIMCOG60	YAV_3/SCO//JO69/CRA/3/YAV79/4/AE.SQUARROSA (498)/5/LINE 1073/6/KAUZ∗2/4/CAR//KAL/BB/3/NAC/5/KAUZ/7/KRONSTAD F2004/8/KAUZ/PASTOR//PBW343	2n = 42	

**Table 2:** List of Chinese applied core germplasm.

Number	Name	Variety Type	No. of Chr	Translocation	Number	Name	Variety Type	No. of Chr	Translocation
CACG1	Daqingmai	landrace	2n = 42		CACG48	Baihuomia	landrace	2n = 42	
CACG2	Guangmai	landrace	2n = 42		CACG49	Sanyuehuang	landrace	2n = 42	
CACG3	Xinkehan9	breeding variety	2n = 42		CACG50	Hongqiangchang	landrace	2n = 42	
CACG4	Kefeng3	breeding variety	2n = 42	2AS.5AS-5AL/5AS.2AS-2AL	CACG51	Quzimai	landrace	2n = 42	
CACG5	Kelao4	breeding variety	2n = 42		CACG52	Pingyuan50	breeding variety	2n = 42	
CACG6	Xinshuguang1	breeding variety	2n = 42	1AS.4BS, 1AL.4BL	CACG53	Baibiansui	landrace	2n = 42	
CACG7	Dongnong101	breeding variety	2n = 42		CACG54	Gejiaxiang	landrace	2n = 42	
CACG8	Xinshuguang6	breeding variety	2n = 42		CACG55	Dachunbaisilengmai	landrace	2n = 42	
CACG9	Jichun1016	breeding variety	2n = 42		CACG56	Zhahong	landrace	2n = 42	
CACG10	Chixiaomai	landrace	2n = 42		CACG57	Motuoxiaomai	landrace	2n = 42	
CACG11	st2422/464	introduced variety	2n = 42		CACG58	Bianbachunmai-6	landrace	2n = 42	
CACG12	Orofen	introduced variety	2n = 42	3BS.4AL, 3BL.4AS	CACG59	Baimangxiaomai	landrace	2n = 42	
CACG13	Nonglin10	introduced variety	2n = 42		CACG60	Wujiangzhuo	landrace	2n = 42	
CACG14		introduced variety	2n = 42		CACG61	Kangdingxiaomai	breeding variety	2n = 42	
CACG15	Early premiun	introduced variety	2n = 42		CACG62	Zangdong4	breeding variety	2n = 42	
CACG16	Triumph	introduced variety	2n = 42		CACG63	Rikaze54	breeding variety	2n = 42	
CACG17	Lovrin	introduced variety	2n = 42	1RS.1BL	CACG64	Rikaze8	breeding variety	2n = 42	
CACG18		introduced variety	2n = 42		CACG65	Yizhimai	landrace	2n = 42	
CACG19	Tanori	introduced variety	2n = 42		CACG66	Dabaimai	landrace	2n = 42	
CACG20	Atlas66	introduced variety	2n = 42		CACG67	Laohan	landrace	2n = 42	
CACG21	Gansu96	breeding variety	2n = 42		CACG68	Huoliyan	landrace	2n = 42	4BS.2AS, 4BL.2AL
CACG22	Chaoanxiaomai	landrace	2n = 42		CACG69	Shanmai	landrace	2n = 42	4AS.4BL, 4AL.4BS
CACG23	Chike	landrace	2n = 42	2BL.4DS, 2BS.4DL	CACG70	Hongtuzi	landrace	2n = 42	
CACG24	Songruimai4	breeding variety	2n = 42		CACG71	Baidatou	landrace	2n = 42	
CACG25	Shengen	landrace	2n = 42		CACG72	Jinhuangmai	landrace	2n = 42	
CACG26	Shanglinxiaomai	landrace	2n = 42		CACG73	Huzhuhong	landrace	2n = 42	
CACG27	Pingyang27	breeding variety	2n = 42		CACG74	Jinmai4	breeding variety	2n = 42	
CACG28	Jinan2	breeding variety	2n = 42	1DL.4AL, 1DS.4AS	CACG75	Dingxi24	breeding variety	2n = 42	
CACG29	Qubao	landrace	2n = 42		CACG76	Ning10	breeding variety	2n = 42	
CACG30	Bainong3217	breeding variety	2n = 42		CACG77	Fan6	breeding variety	2n = 42	
CACG31	Yannong15	breeding variety	2n = 42		CACG78	Guinong10	breeding variety	2n = 42	1RS.1BL
CACG32	Xinong6028	breeding variety	2n = 42		CACG79	Yunmai34	breeding variety	2n = 42	1RS.1BL
CACG33	Jibei2	breeding variety	2n = 42	1RS.1BL	CACG80	Xingyi4	breeding variety	2n = 42	1RS.1BL
CACG34	Neichan5	breeding variety	2n = 42		CACG81	Fengmai11	breeding variety	2n = 42	
CACG35	Zhengzhou6	breeding variety	2n = 42		CACG82	Tongjiabaxiaomai	landrace	2n = 42	
CACG36	Jinan17	breeding variety	2n = 42		CACG83	Honghuamai	landrace	2n = 42	
CACG37	Shannong7859	breeding variety	2n = 42	1RS.1BL	CACG84	Baimaizi	landrace	2n = 42	5BS.2DS, 5BL.2DL
CACG38	Aifeng3	breeding variety	2n = 42		CACG85	Chengduguangtou	landrace	2n = 42	
CACG39	Lumai1	breeding variety	2n = 42	1RS.7DS, 1BL.7DL	CACG86	Jiangmai	landrace	2n = 42	
CACG40	Wenmai6	breeding variety	2n = 42		CACG87	Baihuamai	landrace	2n = 42	
CACG41	Laizhou953	breeding variety	2n = 42	1RS.1BL	CACG88	Huanxiangmai	landrace	2n = 42	
CACG42	Baimangmai	landrace	2n = 42		CACG89	Hanzhongbai	landrace	2n = 42	
CACG43	Huangguaxian	landrace	2n = 42		CACG90	Xiaosanyuehuang	landrace	2n = 42	
CACG44	Banjiemang	landrace	2n = 42		CACG91	Suotiaohongmai	landrace	2n = 42	
CACG45	Quanguding	landrace	2n = 42		CACG92	Hongxumai	landrace	2n = 42	
CACG46	Xishanbiansui	landrace	2n = 42		CACG93	Zimai	landrace	2n = 42	
CACG47	Honggoudou	landrace	2n = 42		CACG94	Chinese Spring	landrace	2n = 42	Reference genome

**Table 3:** Frequencies of different ND-FISH types in the CIMMYT Mexican core germplasm and Chinese applied core germplasm.

Chr	No. of Types	Type	CIMCOG Wheat Lines		CACG Wheat Lines		Chr	No. of Types	Type	CIMCOG Wheat Lines		CACG Wheat Lines		Chr	No. of Types	Type	CIMCOG Wheat Lines		CACG Wheat Lines	
			No. of Lines	Percent (%)	No. of Lines	Percent (%)				No. of Lines	Percent (%)	No. of Lines	Percent (%)				No. of Lines	Percent (%)	No. of Lines	Percent (%)
1A	2	I	48	80.00			1B	8	I	2	3.33			1D	8	I	27	45.00	60	64.52
		II	12	20.00	92	98.92			II	5	8.33	41	44.09			II	21	35.00	6	6.45
2A	13	I	13	21.67					III	35	58.33	8	8.60			III	5	8.33	3	3.23
		II	10	16.67	14	15.05			IV	11	18.33	24	25.81			IV	5	8.33	4	4.30
		III	34	56.67	49	52.69			V			2	2.15			V			16	17.20
		IV	2	3.33	4	4.30			VI			4	4.30			VI			1	1.08
		V	1	1.67	3	3.23			VII			4	4.30			VII			1	1.08
		VI			2	2.15			VIII			2	2.15			VIII			1	1.08
		VII			2	2.15	2B	10	I	6	10.00			2D	5	I	1	1.67		
		VIII			4	4.30			II	19	31.67	5	5.38			II	14	23.33	24	25.81
		IX			4	4.30			III	12	20.00	24	25.81			III	40	66.67	51	54.84
		X			4	4.30			IV	11	18.33	7	7.53			IV	3	5.00	3	3.23
		XI			1	1.08			V	12	20.00	2	2.15			V			14	15.05
		XII			2	2.15			VI			19	20.43	3D	4	I	4	6.67		
		XIII			2	2.15			VII			7	7.53			II	42	70.00	37	39.78
3A	4	I	60	100.00	80	86.02			VIII			8	8.60			III	11	18.33	55	59.14
		II			7	7.53			IX			17	18.28			IV	1	1.67	1	1.08
		III			5	5.38			X			3	3.23	4D	4	I	6	10.00		
		IV			1	1.08	3B	12	I	2	3.33					II	47	78.33	48	51.61
4A	10	I	20	33.33					II	16	26.67	8	8.60			III	5	8.33	43	46.24
		II	24	40.00					III	25	41.67	6	6.45			IV			1	1.08
		III	1	1.67					IV	6	10.00	4	4.30	5D	3	I	50	83.33	17	18.28
		IV	1	1.67					V	3	5.00	21	22.58			II	5	8.33	33	35.48
		V	1	1.67	6	6.45			VI	5	8.33	10	10.75			III	3	5.00	43	46.24
		VI	7	11.67	22	23.66			VII	3	5.00	3	3.23	6D	3	I	58	96.67	77	82.80
		VII	4	6.67	6	6.45			VIII			6	6.45			II			10	10.75
		VIII	2	3.33	24	25.81			IX			3	3.23			III			6	6.45
		IX			31	33.33			X			10	10.75	7D	4	I	4	6.67		
		X			1	1.08			XI			13	13.98			II	46	76.67	81	87.10
5A	9	I	6	10.00					XII			8	8.60			III	8	13.33	10	10.75
		II	4	6.67			4B	5	I	48	80.00	44	47.31			IV			1	1.08
		III	12	20.00	8	8.60			II	10	16.67	37	39.78	5BS/7BS	1	I	5	8.33		
		IV	31	51.67	39	41.94			III	2	3.33	1	1.08	5BL/7BL	4	I	2	3.33		
		V	4	6.67	7	7.53			IV			7	7.53			II	1	1.67		
		VI	3	5.00	22	23.66			V			1	1.08			III	1	1.67		
		VII			6	6.45	5B	6	I	2	3.33					IV	1	1.67		
		VIII			9	9.68			II	36	60.00	30	32.26	1RS/1BL	1	I	7	11.67	7	7.53
		IX			1	1.08			III	15	25.00	23	24.73	1RS.7DS, 1BL.7DL					1	1.08
6A	2	I	46	76.67	31	33.33			IV	1	1.67	2	2.15	2AS.5AS-5AL/5AS.2AS-2AL					1	1.08
		II	14	23.33	62	66.67			V	1	1.67	4	4.30	1AS.4BS, 1AL.4BL					1	1.08
7A	7	I	38	63.33	22	23.66			VI			33	35.48	3BS.4AL, 3BL.4AS					1	1.08
		II	10	16.67	14	15.05	6B	13	I	7	11.67			4BS.2AS, 4BL.2AL					1	1.08
		III	8	13.33	4	4.30			II	1	1.67			4AS.4BL,4AL.4BS					1	1.08
		IV	4	6.67	30	32.26			III	1	1.67			2BL.4DS, 2BS.4DL					1	1.08
		V			15	16.13			IV	31	51.67	20	21.51	5BS.2DS, 5BL.2DL					1	1.08
		VI			3	3.23			V	14	23.33	10	10.75	1DL.4AL, 1DS.4AS					1	1.08
		VII			5	5.38			VI	2	3.33	3	3.23							
									VII	2	3.33	12	12.90							
									VIII	2	3.33	24	25.81							
									IX			7	7.53							
									X			9	9.68							
									XI			6	6.45							
									XII			1	1.08							
									XIII			1	1.08							
							7B	11	I	7	11.67									
									II	3	5.00									
									III	3	5.00									
									IV	20	33.33	5	5.38							
									V	15	25.00	44	47.31							
									VI	1	1.67	10	10.75							
									VII	1	1.67	20	21.51							
									VIII	1	1.67	4	4.30							
									IX	4	6.67	5	5.38							
									X			3	3.23							
									XI			2	2.15							

**Table 4:** Sequences of oligonucleotide probes.

Name of Probe	Sequence and Fluorochrome Label
Oligo-pSc119.2-1	6-FAM-5′CCGTT TTGTG GACTA TTACT CACCG CTTTG GGGTC CCATA GCTAT3′
Oligo-pTa535-1	Tamra-5′AAAAA CTTGA CGCAC GTCAC GTACA AATTG GACAA ACTCT TTCGG AGTAT CAGGG TTTC3′
Oligo-(GAA)_7_	Cy5-5′GAAGAAGAAGAAGAAGAAGAA3′

## Data Availability

Not applicable.
